# Pre-morbidity and COVID-19 disease outcomes in Pakistani population: A cross-sectional study

**DOI:** 10.12669/pjms.38.1.4235

**Published:** 2022

**Authors:** Kaleem Ullah Toori, Muhammad Arsalan Qureshi, Asma Chaudhry

**Affiliations:** 1 Dr. Kaleem Ullah Toori, FRCP (Glasgow). Department of Medicine, KRL Hospital, Islamabad, Pakistan; 2 Dr. Muhammad Arsalan Qureshi (M.B.B.S). Department of Medicine, KRL Hospital, Islamabad, Pakistan; 3 Dr. Asma Chaudhry, MRCP (UK), FCPS General Medicine (Pakistan). Department of Medicine and Endocrinology, Southend University Hospital, Southend-on-Sea, United Kingdom

**Keywords:** Corona Virus Induced Disease (COVID-19), pre-morbidities, disease severity (asymptomatic, mild, moderate and severe), invasive ventilation, mortality

## Abstract

**Objectives:**

To identify association of underlying pre-morbidities with disease severity and mortality in hospitalized patients with Corona virus disease 2019.

**Methods:**

Total 884 COVID RT-PCR positive patients admitted to KRL Hospital Islamabad from April 2020 to August 2020 were included in this cross-sectional study. Pre-morbidities recorded were hypertension, diabetes mellitus, ischemic heart disease, chronic respiratory disease, chronic kidney disease, chronic liver disease, chronic neuro-psychiatric conditions (stroke and depression) and malignancy. Oxygen requirement, requirement of invasive ventilation, and outcome (recovered versus died) was documented. WHO categories for disease severity were used. Demographic profile and symptoms were also noted. SPSS 22 was used for data analysis. Pearson’s Chi square test was used to see association between pre-morbidities and disease severity categories, oxygen requirement, invasive ventilation and outcome. Pearson’s correlation was applied to analyze the correlation between individual pre-morbidities and disease severity categories. P-value < 0.05 was considered statistically significant.

**Results:**

The mean age was 40 ± 12.21 years with 98.5 % being males. Majority patients (74.8%) were asymptomatic. Fever was the most common symptom. Diabetes mellitus and hypertension were the most commonly recorded co-morbidity. Significant correlation (p value < 0.05) was found between the presence of underlying pre-morbidities and disease severity as well as oxygen requirement, requirement of invasive ventilation and mortality.

**Conclusion:**

Results are compatible with worldwide studies and underlying pre-morbidities are convincing risk factors for disease severity and mortality.

## INTRODUCTION

Coronavirus Disease 2019 (COVID-19) caused by the novel severe acute respiratory syndrome coronavirus-2 (SARS-CoV-2) is an emerging pandemic which has caused chaos worldwide. While the first cases of Corona virus emerged in China in 2019, cases started appearing in Pakistan in February 2020 in Karachi and Rawalpindi.^1^ It was declared a global pandemic in March 2020 by World Health Organization (WHO).^2^ The disease presentations are quite heterogeneous. Majority cases are mild and self-limiting. Some people maybe asymptomatic despite carrying corona virus infection. A small proportion of patients tend to develop disease complications fairly rapidly, progressing to acute respiratory distress syndrome (ARDS), multi-organ failure and death. This places a high burden on hospital facilities and intensive care settings.

The status of pre-existing comorbidities in patients and their effect on disease outcomes during COVID-19 illness have been of strong interest. As COVID-19 is an emerging pandemic, research is actively being carried out worldwide in order to identify any marker that may predict morbidity and mortality outcomes. Many pre-morbidities have been identified which occur frequently as well as those predisposing the patients to severe disease with worse outcomes. Chinese studies have been foremost in identifying certain medical conditions like diabetes, hypertension, obesity, cardiovascular diseases, chronic respiratory and neurological illnesses to be quite frequently present in COVID-19 cases.^3^-^7^ Studies also indicate that the presence of one or more comorbidities may be related to severe disease outcomes including intensive care unit (ICU) admission, requirement of invasive ventilation and death.^3^-^7^ A major metanalysis of 34 studies revealed that 42% of obese patients, 40% hypertensive patients, 17% diabetics, 13% with cardiovascular disease, 8% with respiratory disease, 6% with cerebrovascular disease and 4% with malignancy developed severe and fatal outcomes.^3^

As complete knowledge regarding the disease is still unknown, it is necessary to identify factors in high-risk patients in our population. This will certainly help to tailor the treatment with early interventions for high-risk patients in order to minimize case fatalities. The aim of this study was to understand and quantify the risk of underlying co-morbidities associated with contracting COVID-19 and its progression to severe disease in Pakistani population at a tertiary care facility.

## METHODS

We designed this study as hospital-based, observational cross-sectional study. The study was single-centred, conducted at KRL Hospital Islamabad, where a consecutive series of patients from 1^st^ April 2020 to 31^st^ August 2020 were included. Non probability sampling was carried out. A total number of 884 patients were included in study. The sample size calculation was not applicable since there is no prevalence data of the disease as the pandemic is still emerging.^8^ The research was started after approval by the Hospital’s ethical and research committee (Ref: KRL-HI-ERC/Dec20/28, Dated: 31 March, 2020). The study objectives were explained to the patients or their next of kin if the patient was incapacitated and informed consent was obtained

### Inclusion criteria:

All patients with confirmed COVID-19 with positive Reverse transcription polymerase chain reaction (RT-PCR) from nasopharyngeal swab, whether asymptomatic or symptomatic.

All patients who presented with signs and symptoms of COVID-19 were isolated in an isolation facility. Nasopharyngeal swabs were taken for RT-PCR as per interim guidelines of the WHO. Demographic profile, exposure history, signs, symptoms, pre-morbidities and drug histories were obtained. Laboratory and radiological investigations were done as per hospital’s protocol. Pre-morbidities recorded were hypertension, diabetes, ischemic heart disease, chronic respiratory disease, chronic kidney disease (CKD), chronic liver disease (CLD), chronic neuro-psychiatric conditions (patients with stroke and depression) and malignancy. Details were noted regarding oxygen requirement, ICU admissions and patients who were mechanically ventilated. Outcomes were documented as patients who recovered or died. Case severity definitions were as per the interim guidance of WHO.^9^ Asymptomatic (RT-PCR positive but asymptomatic), mild (RT-PCR positive but no hypoxia), moderate (RT-PCR positive with signs of pneumonia and but no severe hypoxia with Spo2 > 90%), severe (signs of severe pneumonia evident with respiratory rate more than 30 breaths/minute or Spo2 < 90% and critical (with acute respiratory distress syndrome [ARDS] or septic shock). We included both, severe and critical patients under severe category.

Statistical analysis was performed using IBM SPSS 22. Continuous variables like age were reported as mean values with standard deviation. Frequency was calculated for categorical variables including gender, disease severity categories, symptoms, pre-morbidities, oxygen requirement, mechanical ventilation and disease outcome. Chi square test of independence was used to calculate the statistical significance of categorical variables. Pearson’s correlation was applied to analyze the correlation between individual pre-morbidities and disease severity categories. Confidence interval of 95 % was presumed. A p value of < 0.05 was taken as statistically significant.

## RESULTS

A total of 884 patients were admitted during the study period. The baseline demographics, symptom severity, pre-morbidity and outcome frequencies are presented in Table-I. The mean age was 40 ± 12.21 years. Only a small minority were females i.e., 1.5 %. Among the symptomatic, the most frequent symptom was fever. Majority had no pre-morbidities (n=746, 84.4%). Amongst the pre-morbidities, the commonest were diabetes (n=76, 8.6%) and hypertension (n=72, 8.1%). Overall, only 8.6 % (n= 76) needed oxygen therapy, 1.1 % (n= 10) required mechanical ventilation and 1.8% (n=16) patients died.

**Table-I:** Baseline characteristics of patients (n=884).

Characteristics	COVID-19 patients
Age (in years) – Mean ± SD	40 ± 12.21
***Gender- n (%)***	
Male	871 (98.5%)
Female	13 (1.5%)
***Disease Severity- n (%)***	
Asymptomatic	661 (74.8%)
Mild	144 (16.3%)
Moderate	37 (4.2%)
Severe	42 (4.8%)
***Symptoms - n (%)***	
Fever	197 (22.3 %)
Dry Cough	123 (13.9 %)
Shortness of breath	71 (8%)
Myalgia	64 (7.2%)
Sore throat	38 (4.3%)
Diarrhea	13 (1.5%)
Headache	7 (0.8%)
Rhinorrhoea	5 (0.6%)
Haemoptysis	1 (0.1%)
Pre-morbidity present - n (%)	n=138 (15.6%)
***Individual Pre-morbidities - n (%)***	
Diabetes Mellitus	76 (8.6%)
Hypertension	72 (8.1%)
Ischemic Heart Disease	26 (2.9%)
Chronic Respiratory Disease	22 (2.5%)
Chronic Kidney Disease	14 (1.6%)
Chronic Liver Disease	4 (0.5%)
Chronic Neuro-psychiatric condition	2 (0.2%)
Malignancy	1 (0.1%)
Required Oxygen Therapy- n (%)	76 (8.6 %)
Required Mechanical Ventilation- n (%)	10 (1.1 %)
***Outcome- n (%)***	
Recovered	868 (98.2 %)
Died	16 (1.8 %)

n = total number of patients.

We analyzed the association of pre-morbidities with disease severity, patients who required oxygen therapy, mechanical ventilation and disease outcome (recovered versus died) as given in Table-II. As evident, the patients who had pre-morbidities had increased disease severity overall and required both oxygen therapy and mechanical ventilation in greater frequency; these results were statistically significant. Amongst the outcome results, the bulk (n=743, 85.6%) of patients who recovered had no pre-morbidities, whereas majority (n=13, 81.3%) of those who died had underlying chronic medical conditions, which was statistically significant (p value < 0.01).

**Table-II:** Association of Pre-morbidities with Disease Severity Categories, Oxygen Requirement, Invasive ventilation requirement and Outcomes - Chi Square Test.

Characteristics	Premorbidities		

	Present	Absent	Chi Square Tests of Independence Pearson χ^2^ value (d.o.f), p- value
***Disease Severity Categories***			
Asymptomatic	47 (7.1 %)	614 (92.9 %)	260.11 ( 3), < 0.01
Mild	31 (21.5 %)	113 (78.5 %)	
Moderate	26 (70.3 %)	11 (29.7 %)	
Severe	34 (81 %)	8 (19 %)	
***Required Oxygen Therapy***			
Yes	59 (77.6 %)	17 (22.4 %)	242.78 (1), < 0.01
No	79 (9.8 %)	729 (90.2 %)	
***Mechanically Ventilated***			
Yes	7 (70 %)	3 (30 %)	22.71 (1) , < 0.01
No	131 (15 %)	743 (85 %)	
***Outcome***			
Recovered	125 (14.4 %)	743 (85.6 %)	53.29 (1), < 0.01
Died	13 (81.3 %)	3 (18.8 %)	

d.o.f.: degree of freedom.

On analysis of the association between individual pre-morbidities and disease severity categories as seen in Table-III, we found significant results of correlation of some parameters with both moderate and severe disease (p-values of both mentioned): diabetes (p-value< 0.01, p-value < 0.01) hypertension (p-value < 0.01, p-value < 0.01), CKD (p-value < 0.01, p-value < 0.01), CLD (p-value < 0.01 with moderate) and chronic respiratory disease (p-value < 0.01, p-value < 0.01). Underlying cardiovascular disease (p-value-0.01) and chronic neurological condition (p-value < 0.01) were significantly correlated with severe disease only. On the other hand, significant negative correlation was established between all pre-morbidities and asymptomatic category, which suggested that asymptomatic patients had no underlying pre-morbidities.

**Table-III:** Correlation of Pre-morbidities with disease severity categories-Pearson’s Correlation.

Premorbidities	Disease Categories			

	Asymptomatic r (p)	Mild r (p)	Moderate r (p)	Severe r (p)
Diabetes	-0.315 ^* *^(< 0.01)	0.044 (0.19)	0.263 ^* *^(< 0.01)	0.317 ^* *^ (< 0.01)
Hypertension	-0.352 ^* *^(< 0.01)	0.070 ^*^(0.03)	0.227 ^* *^(< 0.01)	0.381 ^* *^(< 0.01)
Chronic Kidney Disease	-0.135 ^* *^(< 0.01)	-0.056 (0.09)	0.245 ^* *^(< 0.01)	0.142 ^* *^(< 0.01)
Chronic Liver Disease	-0.116 ^* *^(< 0.01)	0.016 (0.63)	0.154 ^* *^(< 0.01)	0.064 (0.05)
Cardiovascular disease	-0.115^**^(< 0.01)	0.068^*^(0.04)	0.030 (0.36)	0.087^**^(0.01)
Cancer	0.019 (0.56)	-0.015 (0.65)	-0.007 (0.83)	-0.008 (0.82)
Chronic Respiratory Disease	-0.209^**^(< 0.01)	-0.011 (0.73)	0.184^**^(< 0.01)	0.272^**^(< 0.01)
Chronic Neurological Disease	-0.027 (0.41)	-0.021 (0.53)	-0.010 (0.76)	0.101^**^(< 0.01)

r- Pearson correlation coefficient, p- level of significance.

## DISCUSSION

COVID-19 remains a challenge and the rise in cases again over past few months after the initial outbreak has been worrisome. As it’s a novel disease, research is being done actively to identify any factors that may lead to worse outcomes. Pre-morbidities are common and lead to poor outcomes. The mean age in our patients was 40 years which is lower as compared to a Pakistani study where the mean age was 52.58 years.^10^ However as our patient population was quite robust (n=884) compared to the said study^10^ (n=100), it is challenging to compare these study cohorts. Our patients were predominantly males; this is similar to other studies as COVID-19 is known to have a predilection for male gender.^8^,^10^ On the other hand, this was one of our study limitations due to fewer female patients (n=1.5%) being admitted to hospital. The patients in our study were mostly asymptomatic, and the symptomatic ones had fever (n=197, 22.3 %), dry cough (n=123, 13.9 %), shortness of breath (n=71, 8%) and myalgia (n=64, 7.2%) as most common symptoms. This is akin to data acquired by WHO suggesting up to 86% patients maybe asymptomatic and fever indeed is the most common symptom.^11^

We reviewed literature to compare our results of prevalent pre-morbidities found in COVID-19 disease. An American case series based in New York involving 5700 patients revealed hypertension (n=3026, 56.6%), obesity (n=1737, 41.7%) and diabetes (n=1808, 33.8%) as the most prevalent premorbid conditions. They also found that 12.2 % patients’ required mechanical ventilation and 21% died. However, this study did not evaluate the effect of pre-morbidities on disease outcomes.^12^ Similar to our results, a Pakistani study by Asghar et al. found diabetes (n=41/100, 41%) and hypertension (n=32/100, 32%) to be the most prevalent comorbidities in patients with COVID-19, although the frequency was much higher than our study population (diabetes=8.6% and hypertension=8.1%) which might be due to their limited sample size.^10^ The similarity is likely due to comparable metabolic profiles in South Asian ethnicity. Singh et al. conducted a meta-analysis of ten Chinese studies, with chief focus on diabetic cohorts’ association with COVID-19 illness. They concluded that diabetics had a higher incidence (14 – 32 %) of not only severe disease requiring ICU admission and mechanical ventilation, but also a higher mortality (22 – 31%). Other than diabetes, hypertension (21%) and cardiovascular disease (7%) were the main co-morbidities present amongst the COVID-19 pool.^13^

We also reviewed literature to understand the effect of individual pre-morbidities on disease severity outcomes. A major meta-analysis of 34 studies (data of more than 16,000 participants) from the United Kingdom, America, Asian and European countries found that the highest risk (calculated as Odds Ratio) of severe/fatal disease was conferred by chronic respiratory disease (OR 3.56), followed by hypertension (OR 3.17), cardiovascular disease (OR 3.13), kidney disease (OR 3.02), cerebrovascular disease (OR 2.74), malignancy (OR 2.73), diabetes (OR 2.63) and obesity (OR1.72).^3^ Another meta-analysis in China (more than 1500 subjects) revealed that presence of certain comorbidities (COPD, diabetes, hypertension, ischemic heart disease and cerebrovascular disease) is a significant risk factor for disease aggravation requiring ICU admissions.^14^ An interesting Chinese study by Guo et al. looked specifically at association between cardiovascular disease (hypertension and coronary heart disease) and mortality in COVID-19 patients. Patients with underlying cardiovascular disease had a higher need of mechanical ventilation and greater fatality rate.^15^ A major English study utilizing the biobank cohort study (274,356 participants) concluded that specific pre-existing conditions like diabetes, hypertension, coronary heart disease and CKD were independent risk factors for COVID-19 related hospitalizations, severe disease and mortality.^16^ A nationwide analysis in China done by Guan et al. reported that having comorbidities was significantly associated with severe disease outcomes of admission to ICU, requirement of invasive ventilation and death. Outcomes were worse if two or more pre-morbidities co-existed.^8^ We analyzed the pre-morbidities as a whole group and did not analyze the role of individual pre-morbidities in severe/fatal disease outcomes. This can be analyzed in further studies based on strong literature evidence of individual pre-morbidities implicated in severe and fatal outcomes.

Pre-morbidities play a significant role in progression to severe disease, triggering hospital admissions, invasive ventilation and increased fatal outcomes. It is essential for clinicians to advise patients with pre-morbidities to take precautions to prevent the spread of COVID-19 including social distancing, washing hands frequently and using face masks. Interventions are to be focused on individual pre-morbidities. Diabetics are proven to have two to three fold higher risk of hospitalization and death according to a recent Swedish study.^17^ Good glycemic control along with maintaining healthy weight may prevent acquiring severe disease.^17^ Evidence indicates that elderly people with COVID-19 and hypertension are at risk of developing severe illness. Additionally, ACE inhibitors and ARB’s have been shown to be associated with reduced mortality in patients with sepsis. A major Chinese study in 2020 including COVID-19 patients with hypertension found that patients on ACEI/ARB therapy had lower rate of developing severe disease (23.5%) versus non-ACEI/ARB group (48%).^18^ Improved control of hypertension especially with ACEI/ARB is likely to have better outcomes in COVID-19 patients. Another straightforward recommendation for hypertensive patients is offering smoking cessation advice as smoking cessation reduces overall cardiovascular risk as well as severe COVID-19 illness.^19^ British Thoracic Society guidelines recommend chest radiography three months after discharge for all patients admitted to hospital with COVID-19. Patients who suffered severe disease with persisting symptoms or radiological abnormalities require clinical review and further investigation.^20^ Knowledge of these risk factors could help clinicians better identify and manage the high-risk populations.^3^

### Limitations of the study:

The major limitation was that the data relates to employees of a particular institution mostly male hence its findings cannot be generalized for entire population of Pakistan. The study included predominantly male patients as being organization employees, all males were required to be admitted if they tested positive. On the other hand, females which were very few opted to isolate at home and were only admitted if they had moderate to severe disease requiring hospital management. Also, the study was single-centered and over a limited period of four months.

## CONCLUSION

In light of our results and the current literature available, it’s evident that presence of premorbid conditions like diabetes, hypertension, cardiovascular disease, CKD, CLD, chronic respiratory disease and chronic neurological illnesses in COVID -19 patients is linked to having greater disease severity including requirement of invasive ventilation as well as higher probability of fatal outcome. Tailored interventions may be needed in patients who have underlying risk factors. Knowledge of these risk factors could help clinicians better manage the populations at high risk of severe COVID-19.

### Authors’ Contribution:

**KT:** Conceived, designed and did statistical analysis along with review of manuscript. Responsible for the accuracy and integrity of the work.

**MAQ:** Contributed to data collection and manuscript writing.

**AC:** Did manuscript writing with final review of manuscript.

## References

[1] World Health Organization (2020). WHO extends support to Pakistan as it confirms its first two cases of Covid-19[Internet] emro.who.int.com?.

[2] Cucinotta D, Vanelli M (2020). WHO declares COVID-19 a pandemic. Acta Biomed.

[3] Zhou Y, Yang Q, Chi J, Dong B, Lv W, Shen L (2020). Comorbidities and the risk of severe or fatal outcomes associated with coronavirus disease 2019:A systematic review and meta-analysis. Int J Infect Dis.

[4] Chang MC, Park YK, Kim BO, Park D (2020). Risk factors for disease progression in COVID-19 patients. BMC Infect Dis.

[5] Yang J, Zheng Y, Gou X, Pu K, Chen Z, Guo Q (2020). Prevalence of comorbidities and its effects in patients infected with SARS-CoV-2:a systematic review and meta-analysis. Int J Infect Dis.

[6] Gold MS, Sehayek D, Gabrielli S, Zhang X, McCusker C, Ben-Shoshan M (2020). COVID-19 and comorbidities:A systematic review and meta-analysis. Postgrad Med.

[7] Bajgain KT, Badal S, Bajgain BB, Santana MJ (2020). Prevalence of comorbidities among individuals with COVID-19:A rapid review of current literature. Am J Infect Control.

[8] Guan WJ, Liang W-h, Zhao Y, Liang HR, Chen ZS, Li YM (2020). Comorbidity and its impact on 1590 patients with COVID-19 in China:A nationwide analysis. Eur Respir J.

[9] World Health Organization (2020). Clinical management of COVID-19:interim guidance May 2020[Internet] Who.int.com. https://www.who.int/publications/i/item/clinical-management-of-covid-19.

[10] Asghar MS, Kazmi SJH, Khan NA, Akram M, Khan SA, Rasheed U (2020). Clinical Profiles, Characteristics, and Outcomes of the First 100 Admitted COVID-19 Patients in Pakistan:A Single-Center Retrospective Study in a Tertiary Care Hospital of Karachi. Cureus.

[11] World Health Organization (2020). Coronavirus disease (COVID-19):Similarities and differences with influenza[Internet] Who.int.com. https://www.who.int/emergencies/diseases/novel-coronavirus-2019/question-and-answers-hub/q-a-detail/q-a-similarities-and-differences-covid-19-and-influenza.

[12] Richardson S, Hirsch JS, Narasimhan M, Crawford JM, McGinn T, Davidson KW (2020). Presenting Characteristics, Comorbidities, and Outcomes Among 5700 Patients Hospitalized with COVID-19 in the New York City Area. JAMA.

[13] Singh AK, Gupta R, Ghosh A, Misra A (2020). Diabetes in COVID-19:Prevalence, pathophysiology, prognosis and practical considerations. Diabetes Metabol Syndr.

[14] Wang B, Li R, Lu Z, Huang Y (2020). Does comorbidity increase the risk of patients with COVID-19:Evidence from meta-analysis. Aging (Albany NY).

[15] Guo T, Fan Y, Chen M (2020). Cardiovascular implications of fatal outcomes of patients with coronavirus disease 2019 (COVID-19). JAMA Cardiol.

[16] Atkins JL, Masoli JA, Delgado J, Pilling LC, Kuo CL, Kuchel G (2020). Preexisting comorbidities predicting severe COVID-19 in older adults in the UK Biobank community cohort. J Gerontol A Biol Sci Med Sci.

[17] Rawshani A, Kj€olhede EA, Rawshani A, Sattar N, Eeg-Olofsson K, Adiels M (2021). Severe COVID-19 in people with type 1 and type 2 diabetes in Sweden:A nationwide retrospective cohort study. Lancet.

[18] Meng J, Xiao G, Zhang J, He X, Ou M, Bi J, Yang R, Di W, Wang Z, Li Z, Gao H, Liu L, Zhang G (2020). Renin-angiotensin system inhibitors improve the clinical outcomes of COVID-19 patients with hypertension. Emerg Microbes Infect.

[19] Walsh K (2021). COVID-19 and hypertension [Internet]. BMJ Best Practice.

[20] George PM, Barratt S, Desai SR, Devaraj A, Forrest I, Gibbons M (2020). Guidance on respiratory follow up of patients with a clinico-radiological diagnosis of covid-19 pneumonia [Internet]. British Thoracic Society.

